# Adipose tissue in health and disease

**DOI:** 10.1098/rsob.200291

**Published:** 2020-12-09

**Authors:** Innocence Harvey, Anik Boudreau, Jacqueline M. Stephens

**Affiliations:** 1 Adipocyte Biology Laboratory, Pennington Biomedical Research Center, Baton Rouge, LA 70808, USA; 2 Department of Biological Sciences, Louisiana State University, Baton Rouge, LA 70803, USA

**Keywords:** adipose tissue, obesity, insulin resistance, metabolic disease, type 2 diabetes, endocrine organ

## Abstract

Adipose, or fat, tissue (AT) was once considered an inert tissue that primarily existed to store lipids, and was not historically recognized as an important organ in the regulation and maintenance of health. With the rise of obesity and more rigorous research, AT is now recognized as a highly complex metabolic organ involved in a host of important physiological functions, including glucose homeostasis and a multitude of endocrine capabilities. AT dysfunction has been implicated in several disease states, most notably obesity, metabolic syndrome and type 2 diabetes. The study of AT has provided useful insight in developing strategies to combat these highly prevalent metabolic diseases. This review highlights the major functions of adipose tissue and the consequences that can occur when disruption of these functions leads to systemic metabolic dysfunction.

## 1. Introduction

Adipose tissue (AT) is now fully recognized as a metabolically active organ. Historically, AT was thought to provide fuel or insulation to organs, and to serve as a connective tissue. Studies in the last two decades have demonstrated that AT plays a critical role in systemic metabolic health. While AT is indeed the primary site for energy storage in the form of lipid, it is also a major endocrine organ, producing and secreting adipose-tissue-specific hormones known as adipokines. In addition to hormones, fat tissue secretes various forms of genetic material, lipid and proteins that all contribute to its substantial endocrine activity. AT also responds to a variety of circulating metabolites and hormones, including lipids, growth hormone, cortisol, insulin, catecholamines and many others. Moreover, AT is known to be a major metabolic organ, along with the liver and skeletal muscle, critical to maintaining proper glucose homeostasis [1]. Disruption in any one of the three primary functions of adipocytes (lipid storage, endocrine function and responsiveness to insulin) can have major impacts on overall metabolic health. Excess adiposity, or obesity, is a major risk factor in several disease states including type 2 diabetes, cardiovascular disease, hepatic steatosis and at least 13 types of cancers [2–5]. Although research in adipocyte biology and physiology has advanced dramatically in recent years, our understanding of the complex processes governing the role of AT in health and disease is still emerging. This review highlights our knowledge of AT pathologies and how they contribute to metabolic diseases, as well as gaps in our understanding of AT biology that require further study.

## 2. Adipose tissue expansion and development

### 2.1. Cellular growth and development

AT is the primary organ for the storage of lipids. Excessive lipid accumulation results in obesity, also known as excessive adiposity or AT expansion, which is driven by adipocyte hyperplasia and/or hypertrophy. Hyperplasia refers to the formation of new adipocytes from preadipocytes through adipogenesis, a highly complex and tightly regulated process involving many hormones and transcription factors [6], most notably peroxisome proliferator-activated receptor gamma (PPARγ), which is absolutely required for adipocyte differentiation and is considered the master regulator of adipogenesis [7]. Although adipogenesis *in vitro* is well understood, the control of this developmental pathway *in vivo*, in the presence of other tissues and a plethora of circulating factors, is less understood, due in part to the limited methodologies available to study adipogenesis *in vivo*. However, recently developed model systems featuring fluorescent labelling of adipocytes have allowed for a more rigorous *in vivo* assessment of adipogenesis [8–10]. For example, Tang and colleagues were able to detect newly formed adipocytes with the use of the AdipoTrak mouse model and demonstrated that PPARγ agonist treatment enhances adipogenesis *in vivo*, supporting previously established *in vitro* studies [8]*.* Utilization of fluorescence-activated cell sorting and liquid chromatography–tandem mass spectrometry methods has led to the identification of several unique types of adipocyte progenitor cells in adipose tissue, and provided insight into adipocyte origin, development and heterogeneity in both mice and humans [11–13]. Furthermore, other recent technologies such as single-cell RNA sequencing have advanced our comprehension of the genes and processes governing adipocyte commitment of precursors, progenitors and adipocyte stem cells [14,15]. The study of adipogenesis *in vivo* is still an emerging field and we have much left to learn. A recent review provides a detailed overview of the complex nature of adipocytes, as well as other cells within adipose tissue, and highlights the advantages of using of single-cell RNA sequencing in the study of adipocyte biology and development [16]. Further utilization of these new methods will enhance our understanding of overall AT expansion.

It is well established that inhibiting adipogenesis in mice can lead to metabolic dysfunction. For example, loss of PPARγ inhibits adipocyte hyperplasia and total AT accumulation, while promoting adipocyte hypertrophy, insulin resistance and other markers of metabolic dysfunction [17]. PPARγ agonists not only promote adipocyte differentiation [18], but also improve overall glucose homeostasis and metabolic health [19–21]. Deuterium labelling has allowed for further study of adipogenesis in humans *in vivo.* In line with the studies above, there is evidence that PPARγ agonists promote femoral adipocyte differentiation and improve insulin sensitivity in humans [22]. New adipocytes resulting from PPARγ-driven adipogenesis facilitate increased lipid storage in AT and are associated with reduced circulating lipids, enhanced glucose disposal and increased fat oxidation in diabetic patients [8,23]. Notably, this formation of new adipocytes is associated with reduced ectopic lipid storage and a decrease in other markers of metabolic syndrome in patients with fatty liver disease [24,25]. Although PPARγ agonists are highly effective insulin sensitizers for type 2 diabetes treatment, their clinical use has been drastically reduced in recent years due to considerable side effects, including weight gain, fluid retention, congestive heart failure and bone fractures [26–29]. Conversely, there is recent data to suggest that enhanced adipocyte turnover negatively impacts metabolic health [30]. However, as the authors indicated, these data are correlative and it is still unclear whether adipocyte death was a driver of the increased adipogenesis. Clearly, more research needs to be performed in this area.

In contrast to hyperplasia, hypertrophy is the enlargement of individual adipocytes by lipid accumulation. Hypertrophy can occur through uptake of dietary lipids from the circulation, or through the fatty acid synthesis pathway in adipocytes, known as *de novo* lipogenesis (reviewed in [31]). Many rodent studies have suggested that larger adipocytes are a characteristic of metabolic dysfunction [32–34]. This notion is supported by clinical studies reporting that increased adipocyte size is associated with insulin resistance, hepatic steatosis and other markers of metabolic dysfunction [27,28,35]. Similarly, adipocyte volume was higher in patients who did not show improvements in insulin resistance following bariatric surgery [36]. Adipocyte hypertrophy has also been associated with insulin resistance and inflammation in healthy patients who are genetically predisposed to type 2 diabetes [37]. While it is generally accepted that impaired adipogenesis and excessive adipocyte hypertrophy are drivers of insulin resistance in obese states, data from several mouse models indicate that the relationship between fat cell size and metabolic dysfunction is not straightforward, and that changes in metabolic parameters can occur in the absence of altered adipocyte size, and vice versa. For example, ablation of *Siah2*, a ubiquitin ligase, results in obesity and enlarged adipocytes, but preserved insulin sensitivity [38]. Conversely, adipocyte-specific mTORc1 depletion in mice leads to smaller adipocytes accompanied by systemic insulin resistance [39]. Similarly, mice with ectopic expression of nuclear SREBP-1c in adipocytes have overt metabolic dysfunction and lipodystrophy, despite having notably smaller adipocytes when compared to controls [40]. Mice lacking collagen VI have large adipocytes due to uninhibited expansion, but have substantially improved whole-body energy and glucose homeostasis [41]. Also, a mouse model with ectopic expression of endotrophin, a proinflammatory adipokine, in adipocytes displayed increased AT inflammation and fibrosis, as well as systemic metabolic dysfunction, while adipocyte size was unchanged [42]. These data and many other examples make it clear that fat cell size is not an absolute indicator of systemic metabolic health. Overall, a balanced combination of adipocyte hypertrophy and hyperplasia is required for appropriate AT expansion and maintenance of metabolic health.

### 2.2. Extracellular development

**Figure 1.**
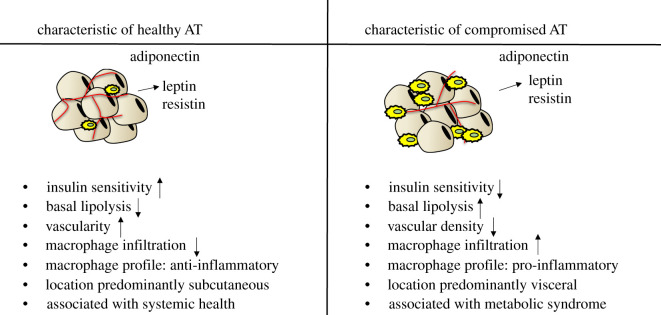
General classifications of metabolically healthy and unhealthy white adipose tissue (AT). Metabolically healthy AT is generally stored subcutaneously, is highly vascularized with low levels of macrophages and has appropriate adiponectin secretion. Healthy adipocytes also have less production and secretion of leptin and resistin. Healthy adipocytes are insulin sensitive with low basal lipolysis that is associated with overall systemic metabolic health. In contrast, metabolically compromised AT is primarily stored in the visceral cavity, has reduced vascularity with increased infiltration or presence of proinflammatory macrophages, and enhanced secretion of leptin and resistin. Typically, unhealthy adipocytes are insulin resistant and have increased basal lipolysis. The increased fatty acids from lipolysis contribute to systemic metabolic dysfunction.

Angiogenesis and vascularization are also important contributors to AT development, as they are required not only for oxygenation, but also for endocrine functions and nutrient transport to and from AT. Insufficient vascularization during AT expansion promotes hypoxia, which may trigger further complications including inflammation, fibrosis and apoptosis [43], contributing to adipose tissue dysfunction (figure 1). While many proteins participate in AT remodelling during expansion, vascular endothelial growth factor (VEGF) is considered the primary player in this process [44]. Several rodent studies demonstrate that reduced AT vascularity in obesity leads to systemic metabolic dysfunction. For example, mice with adipocyte-specific deletion of VEGF that are exposed to high-fat feeding have reduced vascularity, increased inflammation and significantly reduced glucose handling abilities despite a reduction in fat mass [45]. Notably, adipocyte overexpression of VEGF reverses these outcomes [45]. Several review articles have highlighted the implications of impaired VEGF signalling in obesity-induced metabolic disease in humans [46,47]. The anti-angiogenic transcription factor forkhead box O1 (FOXO1) and the angiogenic adipokine neuroregulin 4 (NRG4) are also known to contribute to vascular regulation. FOXO1 levels are elevated in obesity, and mice with reduced endothelial expression of FOXO1 had improved vascular remodelling in AT and enhanced glucose tolerance [48]. However, the FOXO1-deficient mice also had reduced body and fat mass, confounding the interpretation of these findings. NRG4 has recently been recognized as a pro-angiogenic adipokine [49]. Constitutive expression of NRG4 in adipocytes leads to improved glucose tolerance, increased adipose blood vessel formation and reduced hypoxia in AT of obese mice when compared to control mice of the same body weight [49]. Moreover, pharmacological inhibition of angiogenesis or blockade of the NRG4 receptor (ErbB) in the transgenic mice prevented the enhancement of angiogenesis and the favourable metabolic effects noted above [49]. These data suggest that NRG4-induced angiogenesis is a positive regulator of metabolic health in AT. Taken together, these data underscore the notion that AT development as a whole is crucial to systemic health.

## 3. Inflammation

Inflammation is a normal and necessary acute physiological response to a variety of stimuli, such as injury, but is often chronically elevated in several disease states including obesity and metabolic dysfunction. AT inflammation has been extensively studied over the past decade, and obesity is known to be associated with chronic low-grade inflammation and metabolic disease [50–52]. Numerous proinflammatory molecules in AT are involved in obesity-related metabolic disease, including tumour necrosis factor alpha (TNFα), interleukin 6 (IL6), monocyte chemoattractant protein 1 (MCP1) and various adipokines (reviewed in [53], and discussed below in ‘Endocrine functions within adipose tissue’). A high-profile report of obesity-induced inflammation was a study in the early 1990s showing elevated TNFα expression in the AT of genetically obese mice [54]. TNFα is known to induce insulin resistance in adipocytes through several mechanisms, including downregulating the expression of both the insulin receptor and the insulin-sensitive glucose transporter [55,56], as well as impeding insulin signalling events, antagonizing PPARγ action and inducing expression of proinflammatory genes (reviewed in [57]). Conditions such as hyperinsulinaemia and excess circulating lipids result in the recruitment of macrophages and other immune cells to the AT (figure 1), where they act as primary drivers of inflammation through the production of various paracrine factors, including inflammatory cytokines such as TNFα and IL-6 [43,58–62]. In obesity, the chronic overproduction of these inflammatory mediators can cause impaired adipocyte insulin signalling, further inflammation and a continued deterioration of AT function [43,60,63]. Traditionally, macrophages have been classified as either M1 or M2. The M1 type is associated with a proinflammatory environment and enhanced secretion of proinflammatory cytokines from macrophages and adipocytes; whereas the M2 type is considered immunosuppressive and typically plays a more protective or restorative role following inflammatory insults [64]. However, in the last few years, studies have identified several subtypes within the M1/M2 classifications, as well as additional classifications including the newly defined obesity-associated, metabolically activated (MMe) and metabolically oxidized (Mox) macrophages [62,65]. Moreover, new techniques such as single-cell RNA sequencing allows for highly sophisticated analysis of the cellular composition within adipose tissue and has revealed that immune cells represent a substantial percentage of adipose tissue cells and encompass an even greater variety than previously thought [15,66]. The understanding of the contributions of adipose tissue macrophages to adipocyte function and metabolic regulation continues to be an active and expanding area of research.

Inflammasomes, which also contribute to AT inflammation, are also known to influence glucose homeostasis. These multiprotein complexes promote the maturation and secretion of inflammatory cytokines and mediate inflammatory responses to a variety of stress signals, including microbial infection, as well as endogenous mediators such as free fatty acids or extracellular ATP (reviewed in [67]). The most thoroughly characterized is the NLRP3 inflammasome, which is associated with AT inflammation and systemic insulin resistance [67–71]. Its components include a Nod-like receptor (NLR), caspase-1 and apoptosis-associated speck-like protein containing a CARD (ASC) adaptor protein. Whole-body knockouts of inflammasome components have alleviated metabolic disturbances from diet-induced obesity [72,73]; however, these global knockouts also result in reduced body weight and fat mass, complicating the overall interpretation of the metabolic data. More recently, macrophage-specific knockouts have been developed and have yielded similar results [74]. There is evidence to suggest these data are translatable to humans as monocyte-derived macrophages from newly diagnosed type 2 diabetics expressed higher levels of NLRP3, ASC and proinflammatory cytokines, including several interleukins and TNFα, when compared to non-diabetic control macrophages [75]. Moreover, the release of proinflammatory cytokines was significantly elevated in the culture media as well as in the serum of diabetic patients at baseline and following stimulation from fatty acids, when compared to non-diabetic controls [75]. These findings are consistent with previous studies documenting macrophage infiltration into AT as a feature of obesity-associated metabolic dysfunction in mice [59]. While our understanding of AT macrophages and the involvement of other AT immune cells in metabolic health and disease continues to evolve, it is clear that proinflammatory conditions are implicated in the pathology of obesity and associated metabolic disease states. However, in a field that is rapidly changing, it is worth noting that some degree of inflammatory signalling appears to necessary for normal AT function. Two proinflammatory cytokines, TNFα and oncostatin M, are known to be required for proper AT expansion and maintenance of insulin sensitivity in mice [76–79]. Moreover, recent findings suggest that inhibition of a high-fat diet induced inflammation specifically in adipocytes interferes with proper glucose handling [80]. Although AT inflammation clearly has detrimental effects in obesity, it also has adaptive and homeostatic roles in AT expansion and function and its impact on systemic metabolic regulation.

## 4. Location of lipid storage

On the whole, excess adiposity poses an increased risk of developing metabolic syndrome [81] and type 2 diabetes [82,83]. However, there are individuals who have increased adiposity in the absence of metabolic dysfunction, and are considered metabolically healthy obese [84]. Clearly, factors beyond simple adiposity are involved in regulating systemic metabolic homeostasis (figure 1). It is recognized that AT is highly heterogeneous, and that its many functions are impacted by parameters such as its constituent adipocyte types and anatomical locations. First, it is important to understand that there are several different types of adipocytes and AT, each with unique metabolic profiles. These types include white, brown and beige (or ‘brite’) fat. White AT (WAT) is the most abundant, and is the main focus of this review. Its metabolic characteristics are largely dependent on its anatomical location, as discussed below. Brown AT (BAT) is characterized by its ability to generate heat by uncoupling fuel oxidation from ATP generation, a process of metabolic inefficiency that has been speculated to be favourable to weight loss. Beige/brite AT is a newer designation and typically refers to white AT that has acquired some characteristics of brown fat. There is controversy regarding beige fat. While a large body of literature suggests that beige fat is metabolically beneficial [85,86], it has also been considered a stress response to a large variety of conditions [87,88]. The functional differences among the AT varieties are not only dependent on location or energy production and utilization, but also on differences in gene expression, lipid droplet size, innervation and mitochondrial density. BAT and beige AT biology and function have been extensively reviewed [89]. Adding to the complexity of AT biology, recent studies reveal significant heterogeneity *within* the individual AT depots that likely impacts overall function and metabolic health [15,90,91]. Characteristics of AT heterogeneity are an emerging area of investigation; therefore, our understanding is still in its infancy.

The anatomical location of AT also influences systemic metabolism and overall health. Fat depots found in humans are not metabolically or anatomically identical to those found in rodents [92]. This information should be considered when interpreting AT studies. In general, WAT depots are broadly categorized as subcutaneous (located under the skin) or visceral (surrounding internal organs), and these distinctions are widely used and accepted in the study of AT. Typically, subcutaneous AT is considered metabolically healthy, especially when located around the gluteal-femoral region [93]; whereas visceral AT is associated with inflammation and increased metabolic disease risk. Specifically, human clinical studies have reported strong positive correlations between visceral fat and metabolic syndrome components including HOMA-IR and triglycerides, as well as hepatic steatosis, fibrosis and inflammation [94–96]. Moreover, visceral fat is positively associated with cardiovascular disease [95] and inflammatory markers [94,97]. Patients with severe obesity that had an omentectomy (where less than 1% of total fat was removed) in addition to gastric banding had significantly improved glucose handling 2 years after surgery when compared to gastric banding control patients, without significant differences in weight [98]. However, it is important to note that while not statistically significant, the omentectomy group lost more weight and had significantly lower BMI, potentially confounding these data. Metabolic improvement from omental removal has also been observed in lean dogs where omentectomy resulted in enhanced glucose uptake when compared to sham-operated dogs without significant alterations in visceral or total adiposity [99]. Similarly, metabolic improvements are observed when visceral fat is removed from mice, but not from the removal of other AT depots [100]. Conversely, subcutaneous AT is generally positively associated with metabolic health. Findings across several studies indicate that subcutaneous AT is negatively correlated with circulating triglycerides, insulin and glucose in humans [93,101]. This is supported by evidence in obese rodents showing that transplantation of subcutaneous depots from the same mouse or a donor mouse into the visceral cavity improves metabolic profiles without altering total fat mass; however, this was not true of visceral depot transplants [102,103]. Collectively, these data suggest that the location of AT may be a stronger predictor of metabolic health than total fat mass is, and that large amounts of AT in the visceral cavity are detrimental.

One major hypothesis as to why visceral fat has such detrimental effects on metabolic health is its proximity to the portal vein, such that anything released from visceral depots, including fatty acids and inflammatory molecules, have direct access to the liver. This idea is referred to as ‘the portal theory’ (see review [104]). In contrast, subcutaneous fat depots drain into the vena cava and enter the systemic circulation. In support of this theory, one study showed that donor epididymal AT depot transplants into the mesenteric cavity of recipient mice (i.e. portal drainage) resulted in significant glucose intolerance, increased IL-6 expression and macrophage infiltration [105]. On the contrary, when a depot of equal size from the same donor was transplanted to the visceral side of the peritoneum of another littermate (i.e. caval drainage) modest improvements in glucose tolerance were observed. Interestingly, ablation of IL-6 prevented glucose disturbances and reduced other inflammatory markers in the portal-drained group, indicating that inflammatory mediators released from the visceral AT may play a significant role in the associated pathology. Another study in obese rats showed that mesenteric visceral fat, which drains into the portal vein and most closely resembles human visceral AT, played a greater role in insulin resistance when compared to perirenal and epididymal fat depots (typically considered to be visceral depots in rodents, although they do not drain portally) [106]. These data further support AT location as a major driver of metabolic symptoms associated with AT dysfunction.

Though the data described in support of the portal theory are sound and rigorous, there is evidence that glucose disposal rate is negatively associated with the amount of subcutaneous fat, suggesting that subcutaneous depots can also contribute to poor glucose metabolism [107,108]. Intrinsic depot-specific properties, such as inflammatory profiles and lipolytic rates are difficult to separate from the potential impacts of anatomical position (such as portal versus caval drainage), and more research in this area is warranted to determine the relative contributions of these factors to metabolic health. Also, the characteristics of AT that are primarily responsible for improvements in metabolic health are still largely unknown. Hopefully, future research and methods will identify more specific drivers of metabolic health and disease. Nonetheless, we can conclude that both the metabolic profile and the location of fat tissue can substantially contribute to metabolic disease.

Finally, in instances where there is insufficient AT mass or elevated AT lipolysis (discussed in further detail below), lipids can be stored ectopically across several tissues resulting in metabolic dysfunction. This phenomenon is commonly observed in lipoatrophy, a condition in which there is very little AT present, thereby preventing proper lipid storage. As a consequence, individuals with lipoatrophy have elevated circulating lipids, as well as ectopic fat storage in the liver and muscle [109,110], conditions that are commonly associated with impaired glucose homeostasis [111,112]. Indeed, ectopic lipid accumulation in cardiac and skeletal muscle can result in tissue-specific and systemic insulin resistance [113–117]. As a whole, these findings underscore the importance of proper lipid storage within the AT and away from internal organs or skeletal muscles.

## 5. Adipose tissue lipolysis and insulin resistance

Insulin resistance and the progression to type 2 diabetes are among the most common metabolic syndrome co-morbidities associated with obesity [83,118]. AT contributions to systemic insulin resistance have been previously addressed and are discussed throughout this review, but insulin resistance within the AT should also be considered when evaluating the role of AT in metabolic diseases. Although skeletal muscle is responsible for the majority of insulin-stimulated glucose uptake [119], proper insulin signalling in AT is also important for systemic regulation of blood glucose as revealed by a variety of different mouse models. For example, adipocyte-specific ablation of GLUT4, the primary glucose transporter responsible for insulin-stimulated glucose uptake in AT and muscle, impairs insulin signalling in liver and muscle, and induces systemic insulin resistance and glucose intolerance in mice [120]. Likewise, adipocyte-specific insulin receptor knockouts have similar basal glucose uptake, but significantly reduced insulin-stimulated glucose uptake in adipocytes when compared to controls [121]. Notably, these mice have improvements in systemic glucose tolerance and this discrepancy may be a result of an upregulation in other signalling pathways to combat the loss of adipocyte insulin signalling from congenital gene ablation. For instance, one study investigated the effects of insulin receptor (IR) and insulin-like growth factor 1 receptor (IGF-1R) on outcomes of metabolic disease in chow-fed mice by generating adipocyte-specific inducible knockout (KO) models of one (IRKO or IGF-1RKO) or both of these receptors (double KO; DKO [122]). Despite all KO groups having similar or reduced fat mass when compared to controls (depot dependent), the IRKO and DKO mice displayed systemic insulin resistance and hepatic steatosis when compared to the control and IGF-1RKO groups, with the combined deletion of these receptors resulting in the greatest disturbances in glucose handling. These findings suggest that a compensatory mechanism may be activated in other insulin responsive tissues, potentially including non-insulin dependent signalling pathways, to combat systemic glucose intolerance when there are defects in insulin receptor signalling in the AT from birth. Nevertheless, these data indicate that proper insulin signalling within the AT is key for systemic health.

**Figure 2.**
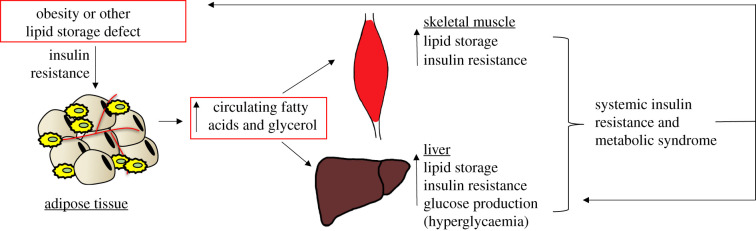
Contribution of adipose tissue dysfunction in the perpetuation of metabolic disease. Disturbances in lipid storage, such as in obesity or lipodystrophy, will interfere with proper adipocyte function and can contribute to insulin resistance. Insulin resistance within the adipose tissue will disrupt normal adipocyte signalling and metabolism resulting in elevated lipolysis. Chronically elevated circulating lipids can lead to ectopic lipid storage and insulin resistance in other tissues, including skeletal muscle and liver. Insulin resistance in the liver in particular is problematic as insulin signalling tightly regulates hepatic glucose production. All of these events can have significant consequences on metabolic health, ultimately resulting in a vicious cycle that perpetuates systemic metabolic disorder.

One way in which AT insulin sensitivity impacts systemic health is through regulation of AT lipolysis (the breakdown of triglycerides into free fatty acids and glycerol). Lipolysis is induced by adrenergic stimulation to mobilize energy stores in conditions such as fasting, exercise and stress. In the fed state, insulin inhibits lipolysis and promotes lipid storage. Disruption of insulin signalling in AT, therefore, can result in elevated basal lipolysis (reviewed in [123]). The chronic low-grade inflammation associated with obesity also contributes to excessive release of lipids by adipocytes, as the inflammatory cytokine TNFα can also induce lipolysis in a manner independent of insulin signalling (reviewed in [124] and [57]). Indeed, obesity and insulin resistance are known to be associated with high basal lipolysis rates (figure 1). The resulting increase in circulating fatty acid levels promotes further metabolic dysfunction through ectopic lipid accumulation, particularly in liver and muscle [123]. The vicious cycle of insulin resistance and elevated basal lipolysis in adipocytes is represented in figure 2. Type 2 diabetes and hepatic lipid accumulation are often observed in conditions associated with elevated basal lipolysis, including Cushing's syndrome [125,126], as well as in conditions of lipoatrophy where there is an excess of circulating lipids [111]. Although AT may not be directly responsible for the majority of whole-body glucose uptake, it is clear that impaired glucose uptake and lipid storage in AT affect other insulin-responsive organs and thus modulate overall systemic health.

## 6. Endocrine functions within adipose tissue

In addition to being highly insulin-responsive, AT also secretes several molecules involved in glucose regulation and metabolic health (figure 1). These molecules, collectively known as adipokines, can be anti- or proinflammatory. Adipokines can act as endocrine regulators, released into the circulation and affecting several other tissues and organs, but can also regulate local signalling in a paracrine or autocrine manner. Several adipokines have now been discovered (reviewed in [127,128] [129]), but we will focus here on the three adipokines that are produced in mouse adipocytes: leptin, resistin and adiponectin. In 1994, leptin was the first adipocyte-derived endocrine hormone to be discovered. Leptin is released from adipocytes proportionally to AT mass and is acutely regulated by fasting [130–132]. In a normal physiological setting, high levels of leptin signal to the brain to cease food intake and, therefore, is known as an anorexigenic hormone [133–136]. An absence of leptin signalling due to genetic mutations in leptin or the leptin receptor leads to severe obesity from hyperphagia in both mice and humans [137], and restoration of signalling will reverse these effects [138,139]. Leptin's anorectic effects, and its ability to rescue obesity in deficient states initially fuelled enthusiasm that leptin would effectively combat obesity. However, leptin is positively correlated with adiposity in humans [140,141], and leptin resistance is a common occurrence in obese states [141,142]. Therefore, leptin treatment in individuals with obesity who exhibit adequate or even elevated leptin levels has not been as beneficial as once hoped. On the contrary, recent findings from two mouse models of reduced leptin expression exposed to high-fat diet suggest that lower levels of leptin during the progression of obesity are protective against weight gain as well as the associated metabolic dysfunction [143]. In fact, a recent review features several studies that support the notion that a reduction in leptin signalling in the context of obesity is associated with weight loss and metabolic improvements [144]. Moreover, leptin has been recognized as a proinflammatory adipokine, so not only is it not beneficial in enhancing weight loss in the general population, it can actually be detrimental to metabolic health when chronically elevated [145]. Collectively these data suggest that low, but sufficient leptin may be beneficial for maintaining metabolic health. Leptin has also been shown to regulate endogenous cortisol production, thereby indirectly modifying glucose homeostasis [146]. These data highlight the importance of leptin production and signalling in the regulation of food intake and body weight.

Resistin is a proinflammatory adipokine, so named for its ability to promote insulin resistance. It was discovered in 2001 in an effort to identify genes suppressed by the PPARγ agonist and antidiabetic drug, rosiglitazone [147]. Interestingly, this endocrine hormone was also identified by another laboratory as an inhibitor of adipogenesis, and named ‘adipocyte-specific secretory factor (ADSF)’. Not surprisingly, given its relationship with insulin resistance, resistin is elevated in obesity in mouse and man [147,148]. Loss of resistin through gene ablation or inactivation improves glucose metabolism in obese mouse models [149,150]. Evidence indicates that resistin enhances the protein levels and activity of SOCS3, which is required for resistin's ability to reduce insulin signalling in adipocytes [151]. Though most of the data surrounding resistin are negative in terms of how it affects metabolic health, there is also evidence that resistin is important in the regulation of fasting blood glucose [152]. Therefore, it is likely that resistin is necessary for glycaemic control, further illustrating the importance of adipocyte endocrine function in sustaining metabolic health. It bears mention that while resistin is primarily secreted from adipocytes in rodents, macrophages are the predominant source of resistin in humans. Nevertheless, resistin's function remains the same across species [153,154].

Adiponectin, an endocrine hormone released by adipocytes, is known to have anti-inflammatory effects and can enhance insulin sensitivity in several tissues, most notably the skeletal muscle and liver. Adiponectin acts via two G protein-coupled receptors called Adipor1 and Adipor2 and highly expressed in muscle, liver and heart [155,156]. In contrast to leptin, adiponectin circulating levels are lower in obesity and type 2 diabetes [157]. Adiponectin exerts its anti-diabetic effects mainly through suppression of hepatic glucose production [158–160], but also enhances glucose uptake in skeletal muscle *in vitro* [161,162]. Administration of adiponectin significantly lowers blood glucose in diabetic mice without affecting insulin levels [158], and has not been shown to induce hypoglycaemia, an added benefit in the treatment of diabetes. Adiponectin can also act in an autocrine manner, as underscored by the fact that it was first discovered in an effort to identify genes involved in adipogenesis [163]. It has now been shown that adiponectin can increase insulin-independent and insulin-stimulated glucose uptake within primary rat adipocytes [164] and regulate lipid accumulation and glucose uptake within the adipocyte [165]. Adiponectin signalling is also important in cardiac muscle, as low hormone levels are associated with coronary artery disease [166]. Furthermore, adiponectin is reported to enhance multiple signalling events including antioxidant, vasodilation and anti-inflammatory activities thought to promote cardiomyocyte health [167,168]. However, it should be noted that there is some controversy regarding adiponectin and cardiac health, as high adiponectin levels have been linked to cardiac dysfunction [169]. Although still not widely recognized, AT is a *bona fide* endocrine organ, releasing hormones and participating in interorgan communication to regulate glucose homeostasis and systemic health.

## 7. Emerging approaches to combat adipose tissue-derived metabolic dysfunction

The studies described in this review highlight the substantial complexities associated with AT in health and disease. As described, alterations in any adipocyte function can be detrimental to overall health. However, as our knowledge of adipocyte biology has expanded, a variety of interventions have emerged as potentially viable therapeutic strategies to ameliorate these metabolic disturbances. Listed below are a few strategies that have recently been investigated to combat adipocyte-mediated contributions to systemic metabolic disease states.

### 7.1. Exercise

Exercise is known to be extremely beneficial for health, and it has been shown to improve glucose homeostasis [170,171]; however, AT-specific effects of exercise have not been studied until recently. There is now evidence that exercise may drive improvements in inflammatory profiles and insulin signalling in AT. Specifically, in a rat model of HFD-induced obesity, aerobic-interval exercise training significantly improved macrophage and inflammatory profiles, as well as capillary density in AT when compared with controls [172]. Moreover, transplantation of WAT from exercise-trained mice into sedentary mice significantly improved systemic glucose tolerance and insulin sensitivity in chow-fed and HFD-fed animals when compared to sham controls or transplantation of sedentary tissue from donor mice given the same diet [53]. Lastly, exercise-trained mice displayed significant elevations in the expression of genes involved in browning in their WAT [173], potentially enhancing energy expenditure and improving overall metabolism. These data suggest that exercise has direct effects on adipocytes that could mitigate the AT dysfunction associated with systemic metabolic perturbations.

### 7.2. microRNAs

microRNAs (miRNAs) are small non-coding RNAs that generally function as inhibitors of genes by binding to their target mRNA transcripts, thereby preventing gene translation and protein expression. These molecules were discovered in 1990, but AT has only recently been identified as a major source of circulating miRNAs [174]. The importance of miRNA expression and activity within the AT is a fairly new topic and still being explored; however, recent evidence indicates that they are crucial for maintenance of adipocyte function. An adipocyte-specific gene knockout of dicer (the enzyme involved in processing miRNAs) results in significant reductions in all WAT depots and severe insulin resistance [175]. Interestingly, with evidence that circulating miRNAs are altered in individuals with obesity and type 2 diabetes, miRNAs are now being considered as potential biomarkers of metabolic health in humans, and are being investigated as potential therapeutics in the treatment of metabolic disease (reviewed here [176]), clearly illustrating miRNAs as promising targets in the regulation of metabolic syndrome.

### 7.3. Exosomes

Exosomes are a particular type of extracellular vesicles that can transport a wide range of materials, including proteins, lipids, metabolites and different species of RNA. In recent years, exosomes have been identified as mediators of disease pathology and as potential therapeutics (reviewed in [177]). Adipose-derived exosomes are currently the subject of intense study, as they are now known to have a critical role in interorgan communication, and to modulate whole-body metabolism (reviewed in [178]). Recent evidence suggests that AT exosomes are significant transporters of circulating miRNAs [174]. A study in diet-induced obese mice showed that intraperitoneal injections of exosomes from isolated adipose-derived stem cells originating from the epididymal WAT of lean mice promoted a shift in macrophage polarization from M1 to M2 and resulted in significant reductions in markers of inflammation within the circulation [179]. Additionally, the administration of exosomes resulted in significant improvements in glucose tolerance, as well as significant reductions in hepatic lipid accumulation. Exosomes are also implicated in paracrine signalling within the AT, providing transport from different cell types and allowing for intra-organ communication. One group reported the surprising finding that caveolin-1 (Cav-1) protein was detected in adipocytes where the *Cav1* gene had been successfully deleted [180]. It was determined that Cav-1 was being transported via exosome from nearby endothelial cells and taken up by the adipocytes. Furthermore, data from this paper also suggest that exosome production in response to stimuli such as the fasting/feeding transition is blunted in obesity. These studies support the use of exosomes as a treatment for metabolic disease. In fact, exosomes are currently being investigated for their ability to package and deliver microRNAs as therapeutics [176].

## 8. Concluding remarks

Novel methodologies and technical advances continue to drive the elucidation of complex mechanisms involved in the contributions of AT to health and disease. We have summarized the principal features of AT function and dysfunction in figure 1. In addition to the many unresolved questions we have discussed in this review, it should be noted that mechanistic data from animal models are largely derived from studies on male rodents, and that sex differences in metabolism and AT function are known to exist in rodents as well as humans [181,182]. Therefore, special emphasis should be placed on the study of sex differences in the context of AT in health and disease in the future. In conclusion, while much remains to be learned about how AT contributes to metabolic disease, there is no question that AT is central to systemic health and that disruption of any of its functions can have substantial impacts.

## Data accessibility

This article has no additional data.
